# Management of Nelson’s Syndrome

**DOI:** 10.3390/medicina58111580

**Published:** 2022-11-02

**Authors:** Athanasios Fountas, Niki Karavitaki

**Affiliations:** 1 Institute of Metabolism and Systems Research, College of Medical and Dental Sciences, University of Birmingham, Birmingham B15 2TT, UK; 2 Centre for Endocrinology, Diabetes and Metabolism, Birmingham Health Partners, Birmingham B15 2TT, UK; 3 Department of Endocrinology, Queen Elizabeth Hospital, University Hospitals Birmingham NHS Foundation Trust, Birmingham B15 2TH, UK

**Keywords:** Nelson’s syndrome, Cushing’s, corticotroph tumour progression, bilateral adrenalectomy, tumour growth, pituitary surgery, radiotherapy, observation

## Abstract

Nelson’s syndrome is a potentially severe condition that may develop in patients with Cushing’s disease treated with bilateral adrenalectomy. Its management can be challenging. Pituitary surgery followed or not by radiotherapy offers the most optimal tumour control, whilst pituitary irradiation alone needs to be considered in cases requiring intervention and are poor surgical candidates. Observation is an option for patients with small lesions, not causing mass effects to vital adjacent structures but close follow-up is required for a timely detection of corticotroph tumour progression and for further treatment if required. To date, no medical therapy has been consistently proven to be effective in Nelson’s syndrome. Pharmacotherapy, however, should be considered when other management approaches have failed. A subset of patients with Nelson’s syndrome may develop further tumour growth after primary treatment, and, in some cases, a truly aggressive tumour behaviour can be demonstrated. In the absence of evidence-based guidance, the management of these cases is individualized and tailored to previously offered treatments. Temozolomide has been used in patients with aggressive Nelson’s with no consistent results. Development of tumour-targeted therapeutic agents are an unmet need for the management of aggressive cases of Nelson’s syndrome.

## 1. Introduction

Nelson’s syndrome is a potentially severe condition that may develop in patients with Cushing’s disease (CD) treated with bilateral adrenalectomy (BLA). Although more than 60 years have passed since the first description of the syndrome by Don Nelson and colleagues [1], definite diagnostic criteria are still lacking. A recent expert consensus recommended that radiological evidence of corticotroph tumour progression or new detection of a radiologically visible pituitary tumour after BLA should be considered as the primary criterion for the definition and diagnosis of NS [given that modern imaging methods (especially MRI) allow earlier detection of tumour growth (even before clinical manifestations become evident)]; hyperpigmentation and progressive increase of plasma ACTH levels after BLA should be considered as non-mandatory secondary criteria for the diagnosis (Table 1) [2]. However, demonstration of corticotroph tumour progression has not been considered a prerequisite for the diagnosis of NS in published series [3,4,5,6,7,8,9,10,11]. Enlargement or identification on imaging of previously non-visible corticotroph tumours may also be detected in patients with CD treated with medical therapy (e.g., adrenal steroidogenesis inhibitors and glucocorticoid receptor antagonist) [12,13,14,15].

The prevalence of NS varies significantly between studies, influenced by the criteria used for its diagnosis, the length of follow-up and possible referral bias of the reporting centres. In a recent systematic review of 36 studies including 1316 CD patients treated with BLA, a mean NS prevalence of 26% at a median follow-up of 7 years was found [16]. Median time from BLA to NS diagnosis was 4 years with the shortest reported interval being 2 months and the longest 39 years; this broad range probably reflects the differences in the criteria used for the definition of NS between studies.

The pathophysiological mechanisms leading to NS have not been as yet elucidated. A suggested hypothesis is that NS represents the natural history of a tumour programmed to behave aggressively from the outset. Others propose that tumour growth is driven by the reduced negative glucocorticoid feedback on the hypothalamus with subsequent increase in corticotropin-releasing hormone (CRH) production [17]. Furthermore, well-established predictive factors for NS development are lacking; high ACTH levels during the first year after bilateral adrenalectomy is the most consistently reported predictive parameter [18].

The long-term outcomes of patients with NS after primary management have been poorly explored. This is due to the limited published series, including small number of patients, their differences in the length of follow-up and the heterogeneity in the criteria defining successful treatment of NS [18]. In addition, in some studies, the interpretation of the results is further complicated by the inclusion in the analyses of tumours already showing progression after their primary management [7,8,19,20,21,22,23,24] and by the lack of information on previous therapies [19,21,22,23,24,25,26,27].

In this review, we will present the available treatment options for patients with NS and their outcomes; we will also discuss the management of the small subset of patients with recurrent/aggressive NS.

## 2. Pituitary Surgery

Surgical removal of the corticotroph tumour is the first line treatment in patients with NS [2]. The transsphenoidal route is the preferred approach, depending mostly on the localisation of the tumour and its growth direction; notably, small tumour size and no extrasellar extension have been associated with long lasting remission [28,29]. However, studies assessing the outcomes of patients with NS managed primarily by surgery are scarce and of small sample size [30,31,32,33,34,35,36,37]. Furthermore, there is heterogeneity in the criteria of NS remission after treatment, whilst, in most studies, a clear definition is not provided [18].

In the largest to date study of NS patients from 13 UK pituitary centres, the 10-year tumour progression-free survival was 80% for those primarily treated with pituitary surgery [11]. Similar results were reported by Zielinski et al. [28] and Xing et al. [38]. In the former study, 10 patients with NS were treated with pituitary surgery between 2000 and 2005 and 2 of them developed tumour growth during a mean period of 45.3 months, whilst in the latter one, in 23 patients offered surgery between 1980 and 1999, corticotroph tumour progression was found in 17.4% during a mean follow-up of 3.6 years. Favourable outcomes have been also reported by Kelly et al. in 7 patients with NS, managed with pituitary surgery between 1978 and 1993 and followed-up for a median period of 17 years; further tumour growth developed in 14.3% of the cases [8]. However, suboptimal results of pituitary surgery in NS patients were found by Kemink et al. [29]. In this study of 15 patients diagnosed with NS between 1969 and 1998, surgery was offered in 6 cases and tumour growth was developed in half of them during a median follow-up of 2.2 years [29].

## 3. Radiotherapy

Radiotherapy is an alternative treatment for NS either alone or after pituitary surgery.

### 3.1. Radiotherapy as Primary Treatment of Nelson’s Syndrome

Pituitary radiotherapy has been used as primary treatment of NS in patients who are not good candidates for surgery, due to tumour location or significant comorbidities. Different radiation techniques have been applied between studies and the outcomes vary. Furthermore, in some studies, the inclusion of cases with already recurrent NS in their analyses makes the interpretation of their results difficult [15,18,22,23].

Conventional fractionated photon-based radiotherapy (CRT) was the method of choice mostly in NS patients diagnosed and managed prior to 2000. However, published data on the efficacy of this approach are extremely limited. In the UK Nelson’s study, the 10-year tumour progression-free survival was 52% for the 22 patients who were offered radiotherapy as primary treatment for their NS (including 19 cases treated with CRT) [11]. It should be mentioned that 4 of these 22 patients had already been previously treated with another course of CRT for their CD (prior to the development of NS), possibly reflecting a more aggressive tumour behaviour. In the study by Espinosa de Los Monteros et al., CRT was offered in 4 patients and resulted in corticotroph tumour shrinkage in 2 and stable size in the remaining 2 patients during a median follow-up of 4.4 years [39]. In a small series by Tran et al., 4 patients were primarily treated with CRT and followed-up for a median period of 3 years; in 50% of them ACTH levels normalised, whilst the other 2 experienced clinical remission (no clear definition for this was provided) [33].

Stereotactic radiosurgery (SRS), including gamma-knife, proton beam and by linear accelerator radiosurgery, has been mainly used in the management of NS during the last 20 years with an increasing frequency. Studies assessing the outcomes of this approach have shown favourable results but their data need to be interpreted with caution given that details on other therapeutic interventions prior to SRS are not provided [9,19,22,23,24,25,27,40,41]. Only 4 studies have evaluated the efficacy of SRS as primary treatment of NS. Graffeo et al. reported corticotroph tumour progression in 16.7% (2/12) of the patients offered SRS [30], whilst Bunevicius et al. reported radiological tumour control in all 5 patients managed by gamma-knife radiosurgery [42]; follow-up duration was not available in both studies. Similar results were found by Vik-Mo et al. and Ganz et al.; none of the NS patients (0/5 and 0/3, respectively) treated with gamma-knife radiosurgery developed tumour growth [20,26]. It should be noted, however, that selection bias and small sample size challenge the significance of these results.

### 3.2. Radiotherapy as Adjuvant Treatment of Nelson’s Syndrome

Adjuvant radiotherapy after pituitary surgery is mainly used in cases of incomplete tumour excision aiming to control further growth. In the UK Nelson’s study, the 10-year tumour progression-free survival was 81% in the patients primarily treated with surgery and radiotherapy [11]. In the second published study assessing the outcomes of this approach, corticotroph tumour growth developed in 14% (1/7) of the cases followed-up for a median period of 17 years [8]. Notably, this patient had an already recurrent tumour. NS remission, defined by ACTH levels < 200 pg/mL two hours after morning glucocorticoid dose and no radiological evidence of residual tumour, was reported in 57% of the patients (4/7). In the remaining 2 cases, ACTH decreased but not to the remission levels; one patient had stable tumour residuum, whilst in the second, no residuum was evident.

## 4. Observation

Imaging surveillance is a management option in patients with NS, mostly considered for those with small tumours, not causing mass effects to vital adjacent structures. However, extremely limited studies have assessed the outcomes of this approach.

In the UK Nelson’s study, the 10-year tumour progression-free survival of patients under surveillance was 51%, and in the majority of the cases, active treatment (surgery, radiotherapy, medical therapy or combination of these) was required [11]. High tumour growth rate has been also reported in a series of 8 conservatively managed NS patients; corticotroph tumour progression developed in 87.5% of the cases followed-up for a median period of 2.5 years [29]. In 6 of these cases, subsequent treatment with pituitary surgery or radiotherapy was offered, whereas in the 7th one, massive pituitary haemorrhage occurred 5 years after NS diagnosis.

## 5. Medical Therapy

Up to date, there is no established medical therapy for the control of ACTH levels and/or the corticotroph tumour in patients with NS. Several types of pharmacotherapy have been proposed but data on their effectiveness are limited and inconclusive (Table 2). However, medical therapy could be an option when other management approaches have failed [43].

### 5.1. Somatostatin Analogues

Normal and tumoural pituitary corticotropic cells express mainly somatostatin receptor subtype 5 and, to a lesser extent, subtype 2 [44]. The hypothesis that somatostatin could reduce ACTH levels in patients with NS was initially evaluated in 1975 with promising results [45]. Octreotide, a first-generation somatostatin analogue, has also been reported to be effective in decreasing ACTH levels and controlling tumour volume, or even reducing it in some cases [46,47,48,49].

The development of pasireotide, a second-generation somatostatin receptor multi-ligand with a stronger binding affinity to receptor subtype 5 than octreotide, has shifted the focus of research to this agent as a potential medical therapy for NS. Pasireotide has been approved as a medical treatment for CD, especially with mild disease, given its effect on inhibiting ACTH secretion and potentially on leading to tumour size reduction [50]. However, pasireotide has been associated with high rates of hyperglycaemia necessitating strict monitoring of glucose metabolism. Data on the efficacy of this agent in patients with NS are extremely scarce. Pasireotide has reduced ACTH levels in some case reports [51,52,53], and it has induced tumour shrinkage in one of them [53]. In a recent case series, 7 patients with NS were treated with pasireotide for a 28-week administration period; reduction of ACTH was reported in all of them, but with no significant change in the tumour volume [54]. Based on the above published data, robust conclusions on the role of pasireotide in the management of patients with NS cannot be drawn.

### 5.2. Dopamine Agonists

Dopamine receptors type 2 are expressed in corticotroph pituitary tumours and dopamine agonists, particularly cabergoline, have been suggested as a second-line, off label medical treatment for CD [50]. Bromocriptine has been shown to reduce the ACTH levels in most [55,56,57,58,59], but not all studies [29,60,61] of patients with NS, whilst cabergoline has been associated with NS remission and tumour resolution [62,63], even in cases where bromocriptine was ineffective [64]. The results of these reports are promising but selection bias and small sample size challenge their validity.

### 5.3. Sodium Valproate

Sodium valproate is an inhibitor of the re-uptake of gamma aminobutyric acid in the hypothalamus leading to decreased CRH release [65]. Due to this action, it has been used as a therapeutic agent for NS aiming to reduce ACTH levels. However, studies assessing the efficacy of long-term administration of sodium valproate in patients with NS have reported inconsistent results; some have shown reduction of ACTH levels [65,66,67,68,69,70], whilst others have not [71,72,73].

### 5.4. Peroxisome Proliferator-Activated Receptor-γ Agonists

Peroxisome proliferator-activated receptor-γ is expressed in normal pituitary corticotrophs and abundantly in corticotropinomas [74]. Heaney et al. demonstrated that rosiglitazone exhibited in vitro anti-proliferative and pro-apoptotic effects in human and murine ACTH-secreting pituitary tumour cells, it effectively prevented the in vivo development of corticotroph tumours and it suppressed ACTH secretion in murine models [74]. However, these favourable actions of rosiglitazone have not been confirmed in NS patients treated with this agent [11,75,76,77,78].

### 5.5. Serotonin Antagonists

Serotonin antagonists, particularly cyproheptadine, have been proposed as a possible pharmacological agent for the suppression of ACTH in patients with NS through a potential hypothalamic action and/or a direct effect on ACTH-secreting tumour cells [55]. Despite the beneficial effects of cyproheptadine in some cases [79,80,81,82,83], its efficacy was not consistently reproduced [29,55,56,84] and its use have been abandoned. Similar results were reported for ketanserin, another serotonin antagonist, which was ineffective in the suppression of ACTH levels in patients with NS [85,86].

## 6. Recurrent and Aggressive Nelson’s Syndrome

Despite primary treatment, in a subset of patients, NS will show further progression. Identification of predictive factors is challenging. In the UK Nelson’s study, complexity of previous treatments for the CD prior to NS diagnosis (possibly reflecting corticotroph adenoma aggressiveness from the outset), predicted tumour behaviour after the NS diagnosis; indeed, the highest risk was found in patients treated with pituitary surgery, radiotherapy and BLA for their CD [11].

The management of these patients is individualized, tailored to previously offered treatments and due to the scarce relevant literature, it is not evidence-based. Studies focusing on the long-term outcomes of patients with recurrent NS are extremely limited. A number of approaches (including surgery, radiotherapy, medical therapy, alone or in combination) have been employed with varying results [8,11,26,27,29,30].

An area raising concern is the reported aggressive course of a small subset of recurrent tumours which continued to show multiple growths, despite various treatment modalities; in these cases, outcomes have been poor and mortality high [8,11,19,23,27,28,29,30,37,87]. Very rarely malignant transformation of the tumour can occur [8,11,27,29,30], with prognostic factors remaining unknown. Temozolomide, an oral alkylating chemotherapeutic agent, has been recommended by the European Society of Endocrinology Clinical Practice Guidelines for the treatment of aggressive pituitary tumours and carcinomas [88]. This agent has been used in cases of aggressive NS and has resulted in biochemical remission and tumour shrinkage in some [89,90] but not all the published cases [11,91].

## 7. Conclusions

The management of NS represents a challenging clinical scenario. Pituitary surgery followed or not by radiotherapy offers the most optimal tumour control, whilst pituitary irradiation alone needs to be considered in cases requiring intervention and are not amenable to surgery. Observation may be offered to patients with small lesions, not causing mass effects to vital adjacent structures but close follow-up is required for a timely detection of corticotroph tumour progression and for further treatment if needed. No medical therapy has been consistently found to be effective in patients with NS. However, it could be an option when other treatments have failed. Despite treatment, a subset of patients with NS may develop further tumour growth and, in some cases, a truly aggressive tumour behaviour can be demonstrated. The management of these patients is individualized and tailored to previously offered treatments. Temozolomide is an option, although robust evidence on its effectiveness is currently lacking. Development of tumour-targeted therapeutic agents are an unmet need for the management of aggressive cases of NS. Further multi-centre studies are needed to facilitate the development of evidence-based management pathways for this challenging condition.

## Figures and Tables

**Table 1:** Diagnostic criteria of Nelson’s syndrome [2].

*Primary criterion*
Radiological evidence of corticotroph tumour progression or new detection on imaging of visible pituitary tumour after bilateral adrenalectomy
*Secondary criteria (non-mandatory)*
Progressive increase of plasma ACTH levels after bilateral adrenalectomy Hyperpigmentation after bilateral adrenalectomy

**Table 2:** Types, mechanism of action and reported efficacy of medical therapy in the management of Nelson’s syndrome.

Agents	Mechanism of Action	Efficacy
Somatostatin analogues (octreotide, pasireotide)	Activation of somatostatin receptor subtype 5 (pasireotide) and subtype 2 (octreotide and pasireotide) leading to suppression of ACTH secretion and potential tumour volume control	Octreotide: case reports demonstrating efficacy in decreasing ACTH levels and controlling/reducing tumour volume Pasireotide: decrease of ACTH levels (case reports—case series)
Dopamine agonists (bromocriptine, cabergoline)	Inhibition of ACTH secretion and tumour volume control by activation of dopamine receptors type 2 in corticotropinoma cells	Bromocriptine: inconsistent results in published studies Cabergoline: decrease of ACTH levels and control/reduction of tumour volume (case reports)
Sodium valproate	Decrease of CRH release by inhibition of gamma aminobutyric acid re-uptake in hypothalamus	Inconsistent results on its efficacy
Peroxisome proliferator-activated receptor-γ agonists (rosiglitazone)	Anti-proliferative and pro-apoptotic effects in human and murine tumoural pituitary ACTH-secreting cells in vitro Prevention of corticotroph tumour development and suppression of ACTH secretion in murine models in vivo	Not effective
Serotonin antagonists (cyproheptadine, ketanserin)	Suppression of ACTH secretion through a possible hypothalamic action and/or a direct effect on ACTH-secreting pituitary tumour cells	Cyproheptadine: not effective in most studies—its use has been abandoned Ketanserin: not effective
